# Procalcitonin, C-Reactive Protein, and Neutrophil Ratio Contribute to the Diagnosis and Prognosis of Severe Acute Pancreatitis

**Published:** 2019-12

**Authors:** Yi LIANG, Xianwei ZHAO, Fanliang MENG

**Affiliations:** 1. Department of Emergency, Affiliated Hospital of Jining Medical University, Jining, P.R. China; 2. Department of Digestive System, Shangdong Institute of Parasitical Disease, Shandong Academy of Medical Science, Jining, P.R. China

**Keywords:** Acute severe pancreatitis, Procalcitonin, C-reactive protein, Neutrophil ratio, Diagnosis

## Abstract

**Background:**

We aimed to explore the significance of procalcitonin (PCT), C-reactive protein (CRP) and neutrophil ratio (N%) in the early diagnosis, treatment, and prognosis of severe acute pancreatitis (SAP).

**Methods:**

A total of 104 patients with SAP (SAP group) and 101 patients with mild acute pancreatitis (MAP) (MAP group) admitted to Affiliated Hospital of Jining Medical University, Jining, China were enrolled. The PCT and CRP in serum were detected by a full-automatic biochemical analyzer, and N% in peripheral blood was measured by a hemocyte analyzer.

**Results:**

The peripheral blood PCT, CRP, and N% in the SAP group were significantly higher than those in the MAP group (*P*<0.001). Multivariate Logistic regression analysis showed that acute physiology and chronic health evaluation II (APACHE II) score, Ranson score, PCT, CRP, and N% were independent risk factors for SAP. The receiver operating characteristic (ROC) curve showed that the area under curve (AUC) of PCT, CRP, and N% in diagnosing SAP were 0.906, 0.840, and 0.834 respectively, while that of combined detection was 0.972. The AUC of PCT, CRP, and N% in diagnosing SAP death were 0.907, 0.900, and 0.894, respectively.

**Conclusions:**

Peripheral blood PCT, CRP, and N% contribute to the diagnosis and prognosis of SAP.

## Introduction

Acute pancreatitis (AP) is an inflammatory disease of exocrine pancreas with rapid onset, fast and uncontrollable progress, which ranges from self-limited disease to severer progressive disease with organ dysfunction and death (1). AP may develop into severe acute pancreatitis (SAP) that is associated with multiple organ failure and systemic inflammatory response syndrome (SIRS). The factors causing SAP are complex and closely related to bad living habits, gallstones, alcohol, etc. (2). It has the characteristics of critical illness, many complications and high fatality rate. Moreover, with the improvement of living standards, its incidence rate is increasing year by year (3). Although the vast majority of SAP patients can be cured without complications, as many as 25% of those hospitalized in ICU for 2 weeks, even after treatment, will suffer from serious complications or even death (4, 5). Therefore, early diagnosis and evaluation of SAP is of great significance for the treatment and prognosis of patients.

Acute physiology and chronic health evaluation II (APACHE II) evaluation system, lipase and amylase are commonly used in the clinical evaluation of AP patients. However, APACHE II evaluation system is relatively complex and is not easy to be widely applied. Lipase and amylase are lack of specificity in evaluating the severity of AP (6, 7). The prognosis of SAP patients depends on the degree of pancreatic necrosis and the severity of multiple system organ failure caused by systemic inflammatory response (8). Local tissue damage is closely related to the balance of inflammatory factors in SAP, so limiting inappropriate pro-inflammatory mediators in circulation is the key to patient recovery (9). C-reactive protein (CRP), a kind of inflammatory factor and a sensitive indicator for chronic inflammation diagnosis, is obviously increased in tissue damage and acute inflammation, being a marker reflecting inflammation (10). Procalcitonin (PCT) level is significantly increased in inflammation, infection, and organ failure, so it can be used as an evaluation index for infection (11). The main component of leukocytes in the peripheral blood of normal people is neutrophils, the ratio of which is significantly increased in inflammation and bacterial infection (12).

There are many studies on PCT, CRP and neutrophil ratio (N%) in SAP (13–15), but the diagnostic value of the combination of the three and its role in prognosis evaluation has been rarely studied. Therefore, in this study, PCT and CRP, and N% in peripheral blood of SAP patients were detected to explore their values in the diagnosis and prognosis evaluation of SAP.

## Materials and Methods

### General data

The medical records of 104 patients with SAP (SAP group) and 101 patients with mild acute pancreatitis (MAP) (MAP group) admitted to our hospital from 2016 to 2019 were recruited in this study. There were 63 males and 41 females in the SAP group, aged 27–81 years with an average age of (59.7±8.9) years. Whereas in the MAP group, there were 58 males and 43 females, aged 26–79 years with an average age of (58.3±8.2) years. Inclusion criteria: SAP and MAP meeting the clinical validation of revised 2012 Atlanta Classification, USA (16); pancreatitis confirmed by MRI and CT imaging; patients with typical abdominal distension and abdominal pain symptoms; serum amylase ≥3 times the upper limit of normal standard; all patients admitted to hospital within 24 hours of onset and receiving symptomatic treatment within 48 hours; patients with complete clinical data. Exclusion criteria: patients who used anti-inflammatory and immunosuppressive drugs in the past month; patients who died within 7 days of admission; patients complicated with rheumatic diseases, other surgical acute abdomen, chronic inflammatory diseases, other acute infectious diseases, coronary heart disease, malignant tumors, severe trauma, tuberculosis and neuropsychiatric diseases.

The study was approved by the Ethics Committee of our hospital. The subjects and their guardians were informed and signed fully informed consent forms.

### Treatment methods

After admission, all patients were given conventional symptomatic treatment (17), including gastrointestinal decompression, antacid, amylase inhibition, fasting, circulation improvement, maintenance of water and electrolyte and acid-base balance, and antibiotics when necessary. Important organ functions were strictly monitored and fluid resuscitation were carried out actively. Pancreatic imaging examination was performed on admission, and 3rd, 5th and 7th day of admission, and symptomatic treatment was performed on problems found.

### Outcome measures

Fasting peripheral blood (3mL) was drawn from the patients at the time of admission and placed in vacuum tubes without anticoagulant and anticoagulant tubes containing Ethylenediamine tetraacetic acid-K2 (EDTA-K2). Next, the blood was centrifuged for 10min with a radius of 10cm and a centrifugal force of 1450(×g) to separate the serum. The CRP and PCT (Chundu Biotechnology Co., Ltd., Wuhan, China, batch no: CDJ-1296C-SJH, CD-0208-LIN) in the serum were detected with Johnson Vitros5600 automatic biochemical-immune analyzer (Johnson & Johnson clinical diagnostics, Inc., NY, USA). The operations were carried out in strict accordance with the instruction manual of the instrument and kit. DxH 800 hematology analyzer (Beckman Coulter, Chaska, MN, USA) was used to measure N% in peripheral blood. These indexes of patients in the SAP group were repeatedly detected on the 3rd, 5th and 7th day of admission. Normal CRP: 0–8 mg/L (18); normal PCT: 0–0.05 ng/ml (19); Normal N%: 60–75% (20).

### Statistical methods

SPSS 20.0 (IBM Corp, Armonk, NY, USA) was used for statistical analysis, and GraphPad Prism 6 was used to plot the figures. The counting data were expressed by the number of cases/percentage [N(%)] and the chi-square test was used for comparison between groups. Measurement data accorded with normal distribution were expressed by mean±standard deviation (x¯±SD) and independent samples *t*-test was used for the comparison between groups. Measurement data not accorded with normal distribution were expressed by M (P25, P75) and Mann-Whitney U test was used for the comparison between groups. The data between different time points were compared by repeated measures analysis of variance (ANOVA), and Bonferroni method was used for pairwise comparison between different time points in the group. The receiver operating characteristic (ROC) curve was plotted and the optimal Youden index-based cutoff point was selected to evaluate the diagnostic value of CRP, PCT, and N% in SAP, and to determine their area under curve (AUC), sensitivity, and specificity. The difference was statistically significant with *P* < 0.05.

## Results

### Baseline data

There was no significant difference between the SAP group and the MAP group in sex, age, body mass index (BMI), smoking and drinking history, diabetes mellitus, pathogenic factors, blood and urine amylase, platelet, leukocyte, total cholesterol (TC), high density lipoprotein (HDL), blood urea nitrogen (BUN), alanine aminotransferase (ALT), aspartate aminotransferase (AST), and total bilirubin (TB), while there were significant differences in APACHE-II score and Ranson score (*P* < 0.001) (Table 1).

**Table 1:** Baseline data in SAP group and MAP group [n(%)]/(x¯±SD)

***Classification***	***SAP group (n=104)***	***MAP group (n=101)***	**Z/t/*χ^2^***	**P**
Sex			0.210	0.647
Male	63 (60.58)	58 (57.43)		
Female	41 (39.42)	43 (42.57)		
Age (years)	59.7±8.9	59.7±8.9	1.170	0.243
BMI (kg/m^2^)	22.84±2.14	22.76±2.19	0.264	0.792
Smoking history			0.371	0.542
Yes	44 (42.31)	47 (46.53)		
No	60 (57.69)	54 (53.47)		
Drinking history			0.358	0.550
Yes	46 (44.23)	50 (49.50)		
No	58 (55.77)	51 (50.50)		
Diabetes mellitus			3.185	0.074
Yes	22 (21.15)	12 (11.88)		
No	82 (78.85)	89 (88.12)		
APACHE-II score	10.8±3.2	6.9±3.1	8.859	<0.001
Ranson score	2.0±0.7	1.6±0.9	3.558	<0.001
Pathogenic factors			3.524	0.620
Cholecystolithiasis	40 (38.46)	35 (34.65)		
Choledocholithiasis	11 (10.58)	10 (9.90)		
Overeating	4 (3.85)	2 (1.99)		
Cholecystitis	14 (13.46)	23 (22.77)		
Alcoholic	26 (25.00)	24 (23.76)		
Hyperlipemia	9 (8.65)	7 (6.93)		
Blood amylase (U/L)	977.89 (510.36–1663.16)	967.20 (535.56–1433.15)	−0.403	0.687
Urine amylase (U/L)	2798.40 (1426.59–4024.48)	2404.20 (1067.02–4010.66)	−1.286	0.199
Platelet (×10^9^/L)	150.18 (108.06–194.77)	152.80 (112.49–184.81)	−0.291	0.771
Leukocyte (×10^9^/L)	15.83±5.94	15.23±4.74	0.798	0.426
TC (mmol/L)	3.21±1.02	3.34±1.35	0.779	0.437
HDL (mg/L)	0.91±0.38	0.89±0.22	0.459	0.646
BUN (mmol/L)	13.64 (6.70–19.38)	12.04 (7.53–15.72)	−1.308	0.191
ALT (U/L)	55.01 (30.92–83.99)	55.71 (39.16–74.48)	−0.148	0.882
AST (U/L)	80.48 (50.46–122.34)	78.48 (49.57–110.28)	−0.820	0.412
TB (μmol/L)	45.44 (79.80–62.20)	41.07 (28.86–53.26)	−1.717	0.086

### PCT, CRP, and N% in peripheral blood

The detection showed that the PCT, CRP, and N% in the SAP group were significantly higher than those in the MAP group (*P* < 0.001). Taking baseline data and serological indicators as independent variables, and SAP as a dependent variable, multivariate Logistic regression analysis showed that APACHE II score (*P*=0.001), Ranson score (*P*=0.012), PCT (*P*=0.012), CRP (*P*=0.001), and N% (*P*=0.001) were independent risk factors for SAP (Table 2–3).

**Table 2:** Comparison of peripheral blood PCT, CRP, and N% (x¯±SD)

***Group***	***n***	***PCT (ng/mL)***	***CRP (mg/L)***	***N%***
SAP group	104	1.99±0.25	53.27±16.16	86.09±0.68
MAP group	101	1.56±0.21	32.44±11.68	85.03±0.78
*t*	-	13.650	10.560	10.380
*P*	-	<0.001	<0.001	<0.001

**Table 3:** Multvariate Logistic regression analysis of SAP

***Variable***	***B***	***S.E***	***Wals***	***P***	***OR***	***95% CI***
APACH E-II score	0.291	0.071	16.799	0.001	1.338	1.164–1.538
Ranson score	0.651	0.259	6.315	0.012	1.917	1.154–3.184
PCT (ng/mL)	1.929	0.764	6.383	0.012	6.883	1.541–30.743
CRP (mg/L)	0.128	0.024	28.520	0.001	1.136	1.084–1.191
N%	2.290	0.474	23.375	0.001	9.873	3.902–24.978

### Diagnostic value of PCT, CRP, and N% in SAP

**Fig. 1:**
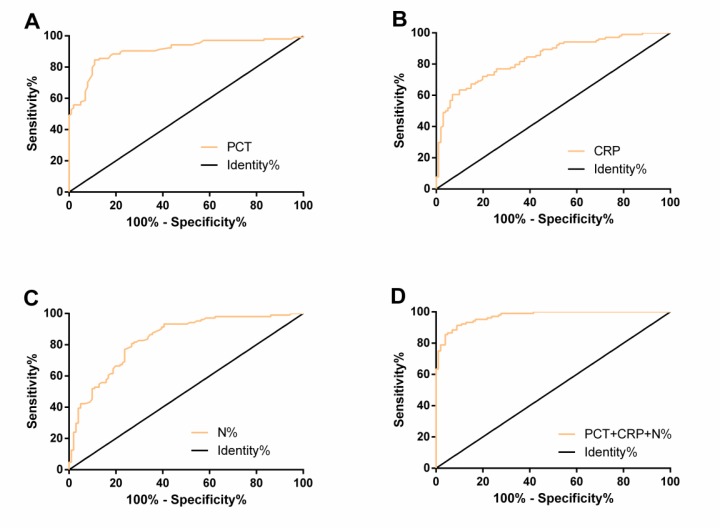
ROC curves of peripheral blood PCT (A), CRP (B), N% (C), and their combination (D) in diagnosing SAP

The ROC curves of peripheral blood PCT, CRP, and N% in diagnosing SAP were plotted. The AUC, cut-off, sensitivity, and specificity of PCT were 0.906, 1.80ng/mL, 84.62%, and 89.11% respectively, those of CRP were 0.840, 51.38mg/L, 60.58%, and 93.07%, and those of N% were 0.834, 85.52%, 80.77%, and 73.27% respectively. Using PCT, CRP, and N% as independent variables, a Logistic regression model was obtained: Logit (P) =−207.022+6.945 PCT+0.090 CRP+2.235 N%. The AUC, sensitivity, and specificity of combined detection of PCT, CRP, and N% in diagnosing SAP were 0.972, 91.35%, and 91.09% respectively (Table 4 and Fig. 1).

**Table 4:** Diagnostic value of PCT, CRP, and N% in SAP

***Index***	***AUC***	***95%CI***	***Std. Error***	***Cut-off***	***Sensitivity (%)***	***Specificity (%)***
PCT (ng/mL)	0.906	0.863–0.948	0.022	1.80	84.62	89.11
CRP (mg/L)	0.840	0.786–0.893	0.027	51.38	60.58	93.07
N%	0.834	0.779–0.889	0.028	85.52	80.77	73.27
PCT+CRP+N%	0.972	0.954–0.990	0.009	0.48	91.35	91.09

### Correlation of PCT, CRP, and N% with APACHE-II score and Ranson score in SAP

**Fig. 2:**
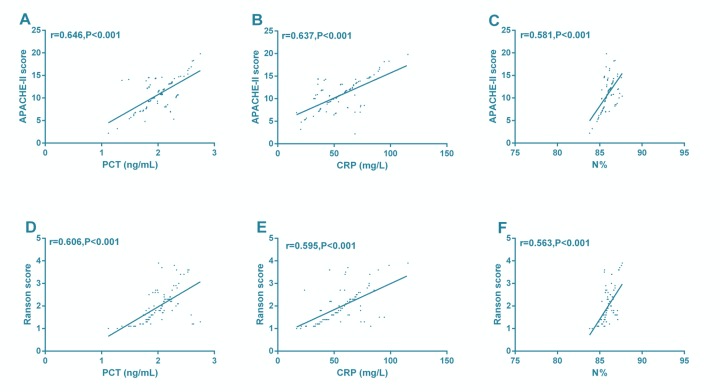
Correlations of PCT, CRP, and N% with APACHE-II score and Ranson score in SAP. PCT (A), CRP (B), and N% (C) were positively correlated with APACHE-II scor. PCT (D), CRP(E), and N% (F) were positively correlated with Ranson score

Pearson correlation coefficient showed that PCT, CRP, and N% were positively correlated with APACHE-II score and Ranson score (*P*< 0.001) (Fig. 2).

### Changes of PCT, CRP and N% at different time points after admission

**Fig. 3:**
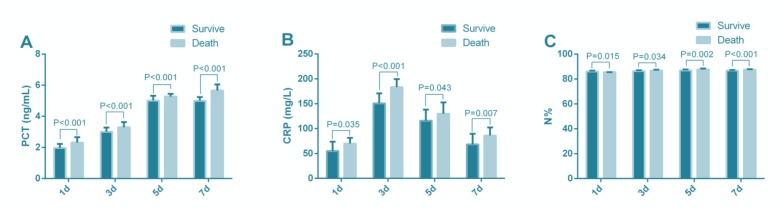
Changes of peripheral blood PCT (A), CRP (B) and N% (C) at different time points after admission

Of 104 patients with SAP, 92 were cured, and 12 died, while all of 101 patients with MAP were cured and discharged from hospital. Repeated measures ANOVA showed that there were significant differences in peripheral blood PCT, CRP, and N% between survivors and deaths at different time points (*P*< 0.001). PCT, CRP and N% in deaths were significantly higher than those in survivors at different time points (*P*<0.05). PCT and N% showed the most obvious difference on the 7th day of admission, while CRP on the 3rd day (Fig. 3).

### Diagnostic value of PCT, CRP, and N% in the prognosis of SAP

**Fig. 4:**
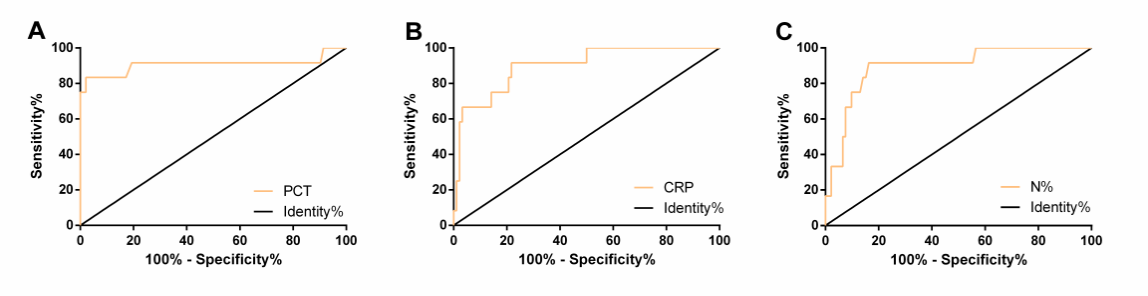
ROC curves of peripheral blood PCT (A), CRP (B), and N% (C) in diagnosing SAP death

ROC curves, with the most obvious statistical difference within 7 days of admission, of PCT, CRP and N% in diagnosing SAP death were plotted. The AUC, cut-off, sensitivity and specificity of PCT were 0.907, 5.52ng/mL, 83.33% and 97.85% respectively, those of CRP were 0.900, 165.30mg/L, 91.67% and 78.26%, and those of N% were 0.894, 87.27%, 91.67% and 83.70% respectively (Table 5 and Fig. 4).

**Table 5:** Diagnostic value of PCT, CRP, and N% in SAP death

***Index***	***AUC***	***95%CI***	***Std. Error***	***Cut-off***	***Sensitivity (%)***	***Specificity (%)***
PCT (ng/mL)	0.907	0.765–1.050	0.073	5.52	83.33	97.85
CRP (mg/L)	0.900	0.812–0.987	0.045	165.30	91.67	78.26
N%	0.894	0.803–0.984	0.046	87.27	91.67	83.70

## Discussion

SAP, a common clinical critical illness with an increasing incidence, has become one of the factors threatening human health (21). Its pathogenesis is considered to be pathological changes of abnormal activated pancreatins that induce monocytes and macrophages to release inflammatory mediators, thus damaging various organs and inducing SIRS (22). The vast majority of SAP patients suffer from necrotizing pancreatitis, and are easily complicated with infection, organ dysfunction, and even death in severe cases (23).

In this study, the PCT, CRP, and N% in the SAP group were significantly higher than those in the MAP group. Logistic regression analysis showed that APACHE II score, Ranson score, PCT, CRP, and N% were independent risk factors for SAP. Therefore, high PCT, CRP, and N% levels increase the risk of SAP. Previous studies have confirmed that APACHE-II score and Ranson score are useful tools for evaluating the severity and prognosis of AP (24, 25). Pearson correlation coefficient showed that PCT, CRP, and N% were positively correlated with APACHE-II score and Ranson score, suggesting that they are expected to be markers for the evaluation of SAP. ROC curves showed that the AUC of PCT, CRP, and N% in diagnosing SAP were 0.906, 0.840, and 0.834, respectively, indicating that they had good diagnostic value for SAP. In the research of Dias et al., plasma PCT and CRP levels were related to the total white blood cell count, as well as the ICU stay and the progress of SAP. During antibiotic treatment, The sensitivity and specificity of PCT were higher in diagnosing SAP when cut-off > 2 ng/mL, while those of CRP were 70% and 65% respectively when cut-off > 19 mg/dL (26). In this study, the optimal cut-off of PCT and CRP was 1.80 ng/mL and 51.38 mg/L respectively. It may be that we detected PCT and CRP in patients with SAP at admission, leading to the different cut-off, sensitivity and specificity. The ROC curve showed that the AUC of combined diagnosis of PCT, CRP, and N% in SAP was 0.972, which indicated that combined detection of the three improved their diagnostic efficiency for SAP. A study has revealed that interleukin-6 (IL-6), IL-8 and other inflammatory factors are indicators to evaluate the severity of early AP (27). Previous studies have confirmed that PCT, CRP, and N% are systemic inflammatory response markers (28, 29). The pathophysiological process of AP is excessive release of inflammatory cytokines and inflammatory transmitters, resulting in severe local tissue damage and systemic inflammation (30). PCT, CRP, and N% may therefore be involved in the course of the AP.

According to a study, inflammatory cytokines measured on admission and CRP measured on the 3rd day of admission could predict systemic complications in patients with AP (31). IL-6 and CRP contributed to early prediction and severity assessment of AP (32). However, there is no single indicator for death monitoring in patients with SAP. We observed the changes of PCT, CRP, and N% in peripheral blood within 7 days, and the results showed that the PCT, CRP, and N% in the deaths were significantly higher than those in survivors at different time points, which indicated that observation of PCT, CRP and N% in peripheral blood was helpful to monitor the prognosis of SAP patients. In Rau et al’s study, monitoring PCT could evaluate the overall prognosis of AP patients with pancreatic infection and SAP patients (33). CRP level of SAP patients reached a peak at 48h after admission, then gradually decreased with time (34). Neutrophillymphocyte ratio could predict the severity of AP and was a risk factor for ICU admission and hospital stay of patients (35). Therefore, PCT, CRP and N% may play important roles in the poor prognosis of SAP, but diagnostic value of them in the SAP death has hardly been studied. The ROC curves of peripheral blood PCT, CRP, and N% in diagnosing SAP death was assessed. Time points with the most statistical difference between the survivors and the deaths were selected. The AUC of CRP in diagnosing SAP death was 0.900 on the 3rd day of admission, while that of PCT and N% was 0.907 and 0.894 respectively on the 7th day. Therefore, detection of PCT, CRP, and N% has good predictive value for poor prognosis of SAP patients.

However, there are still some deficiencies in our study. Firstly, the correlation of PCT, CRP and N% with organ failure and infection in patients with SAP is not explored. Secondly, the risk factors for prognosis (organ failure, infection, death) in patients with SAP are not analyzed. The above deficiencies will be addressed in future studies in order to further support the research conclusion.

## Conclusion

Peripheral blood PCT, CRP and N% contribute to the diagnosis and prognosis of SAP.

## Ethical considerations

Ethical issues (Including plagiarism, informed consent, misconduct, data fabrication and/or falsification, double publication and/or submission, redundancy, etc.) have been completely observed by the authors.

## References

[1] Deng LH Hu C Cai WH (2017). Plasma cytokines can help to identify the development of severe acute pancreatitis on admission. Medicine (Baltimore), 96(28): e7312. 28700471 10.1097/MD.0000000000007312 PMC5515743

[2] Portelli M Jones CD (2017). Severe acute pancreatitis: pathogenesis, diagnosis and surgical management. Hepatobiliary Pancreat Dis Int, 16: 155–159. 28381378 10.1016/s1499-3872(16)60163-7

[3] Otsuki M Takeda K Matsuno S (2013). Criteria for the diagnosis and severity stratification of acute pancreatitis. World J Gastroenterol, 19: 5798–5805. 24124324 10.3748/wjg.v19.i35.5798 PMC3793134

[4] Wang GJ Gao CF Wei D Wang C Ding SQ (2009). Acute pancreatitis: etiology and common pathogenesis. World J Gastroenterol, 15: 1427–1430. 19322914 10.3748/wjg.15.1427 PMC2665136

[5] Swaroop VS Chari ST Clain JE (2004). Severe acute pancreatitis. JAMA, 291: 2865–2868. 15199038 10.1001/jama.291.23.2865

[6] Yang L Liu J Xing Y Du L Chen J Liu X Hao J (2016). Comparison of BISAP, Ranson, MCTSI, and APACHE II in predicting severity and prognoses of hyperlipidemic acute pancreatitis in Chinese patients. Gastroenterol Res Pract, 2016: 1834256. 27882045 10.1155/2016/1834256 PMC5110880

[7] Ismail OZ Bhayana V (2017). Lipase or amylase for the diagnosis of acute pancreatitis? Clin Biochem, 50: 1275–1280. 28720341 10.1016/j.clinbiochem.2017.07.003

[8] Chen YJ Zhuang YD Cai Z (2019). Effects of enteral nutrition on pro-inflammatory factors and intestinal barrier function in patients with acute severe pancreatitis. Eur J Inflamm, 17: 2058739219827212.

[9] Bereanu AS Sava M (2015). Correlation between Intra-Abdominal Pressure and C- Reactive Protein in Acute Pancreatitis. Acta Medica Transilvanica, 20: 109–113.

[10] Liu T Huang W Szatmary P (2017). Accuracy of circulating histones in predicting persistent organ failure and mortality in patients with acute pancreatitis. Br J Surg, 104: 1215–1225. 28436602 10.1002/bjs.10538 PMC7938821

[11] Simsek O Kocael A Kocael P (2018). Inflammatory mediators in the diagnosis and treatment of acute pancreatitis: pentraxin-3, procalcitonin and myeloperoxidase. Arch Med Sci, 14: 288–296. 29593801 10.5114/aoms.2016.57886 PMC5868652

[12] Yang Z Meng X Xu P (2015). Central role of neutrophil in the pathogenesis of severe acute pancreatitis. J Cell Mol Med, 19: 2513–2520. 26249268 10.1111/jcmm.12639 PMC4627557

[13] Staubli SM Oertli D Nebiker CA (2015). Laboratory markers predicting severity of acute pancreatitis. Crit Rev Clin Lab Sci, 52: 273–283. 26173077 10.3109/10408363.2015.1051659

[14] Zheng W Zhang L Long G Chen B Shu X Jiang M (2018). Amalgamation of systemic inflammatory response syndrome score with C-reactive protein level in evaluating acute pancreatitis severity in children. Scand J Gastroenterol, 53: 755–759. 29644912 10.1080/00365521.2018.1459825

[15] Merza M Hartman H Rahman M (2015). Neutrophil extracellular traps induce trypsin activation, inflammation, and tissue damage in mice with severe acute pancreatitis. Gastroenterology, 149: 1920–1931.e8. 26302488 10.1053/j.gastro.2015.08.026

[16] Ignatavicius P Gulla A Cernauskis K Barauskas G Dambrauskas Z (2017). How severe is moderately severe acute pancreatitis? Clinical validation of revised 2012 Atlanta Classification. World J Gastroenterol, 23: 7785–7790. 29209119 10.3748/wjg.v23.i43.7785 PMC5703938

[17] Tenner S Baillie J DeWitt J Vege SS (2013). American College of Gastroenterology guideline: management of acute pancreatitis. Am J Gastroenterol, 108: 1400–1415. 23896955 10.1038/ajg.2013.218

[18] Dashti R Al Jarallah M Rajan R Al Mulla K Khalil M Sayed W (2018). Ruptured mitral valve abscess with mitral incompetence in culture negative infective endocarditis: case report. Eur Heart J Case Rep, 2: yty003. 31020085 10.1093/ehjcr/yty003 PMC6426010

[19] Jeong DK Lee HW Kwon YM (2015). Clinical value of procalcitonin in patients with spinal infection. J Korean Neurosurg Soc, 58: 271–275. 26539272 10.3340/jkns.2015.58.3.271 PMC4630360

[20] Moukarbel GV Yu ZF Dickstein K (2014). The impact of kidney function on outcomes following high risk myocardial infarction: findings from 27 610 patients. Eur J Heart Fail, 16: 289–299. 24464979 10.1002/ejhf.11

[21] Ji L Lv JC Song ZF Jiang MT Li L Sun B (2016). Risk factors of infected pancreatic necrosis secondary to severe acute pancreatitis. Hepatobiliary Pancreat Dis Int, 15: 428–433. 27498584 10.1016/s1499-3872(15)60043-1

[22] Yang R Tenhunen J Tonnessen TI (2017). HMGB1 and histones play a significant role in inducing systemic inflammation and multiple organ dysfunctions in severe acute pancreatitis. Int J Inflam, 2017: 1817564. 28316860 10.1155/2017/1817564 PMC5339498

[23] Maheshwari R Subramanian RM (2016). Severe acute pancreatitis and necrotizing pancreatitis. Crit Care Clin, 32: 279–290. 27016168 10.1016/j.ccc.2015.12.006

[24] Cho J H Kim T N Chung H H Kim KH (2015). Comparison of scoring systems in predicting the severity of acute pancreatitis. World J Gastroenterol, 21: 2387–94. 25741146 10.3748/wjg.v21.i8.2387 PMC4342915

[25] Harshit Kumar A Singh Griwan M (2017). A comparison of APACHE II, BISAP, Ranson’s score and modified CTSI in predicting the severity of acute pancreatitis based on the 2012 revised Atlanta Classification. Gastroenterol Rep (Oxf), 6: 127–131. 29780601 10.1093/gastro/gox029 PMC5952961

[26] Dias BH Rozario AP Olakkengil SA (2015), VA. Procalcitonin strip test as an independent predictor in acute pancreatitis. Indian J Surg, 77: 1012–1017. 27011501 10.1007/s12262-014-1112-8 PMC4775672

[27] Frossard J L Hadengue A Pastor C M (2001). New serum markers for the detection of severe acute pancreatitis in humans. Am J Respir Crit Care Med, 164: 162–170. 11435255 10.1164/ajrccm.164.1.2008026

[28] Gao N Yan C Zhang G (2018). Changes of serum procalcitonin (PCT), C-reactive protein (CRP), interleukin-17 (IL-17), Interleukin-6 (IL-6), high mobility group protein-B1 (HMGB1) and D-dimer in patients with Severe acute pancreatitis treated with continuous renal replacement therapy (CRRT) and its clinical significance. Med Sci Monit, 24: 5881. 30136704 10.12659/MSM.910099 PMC6118162

[29] Jeon T J Park J Y (2017). Clinical significance of the neutrophil-lymphocyte ratio as an early predictive marker for adverse outcomes in patients with acute pancreatitis. World J Gastroenterol, 23: 3883–89. 28638228 10.3748/wjg.v23.i21.3883 PMC5467074

[30] Zhang XP Zhang J Song QL Chen HQ (2007). Mechanism of acute pancreatitis complicated with injury of intestinal mucosa barrier. J Zhejiang Univ Sci B, 8: 888–895. 18257123 10.1631/jzus.2007.B0888 PMC2100161

[31] Fisic E Poropat G Bilic-Zulle L Licul V Milic S Stimac D (2013). The role of IL-6, 8, and 10, sTNFr, CRP, and pancreatic elastase in the prediction of systemic complications in patients with acute pancreatitis. Gastroenterol Res Pract, 2013: 282645. 23476635 10.1155/2013/282645 PMC3583135

[32] Khanna AK Meher S Prakash S (2013). Comparison of Ranson, Glasgow, MOSS, SIRS, BISAP, APACHE-II, CTSI Scores, IL-6, CRP, and procalcitonin in predicting severity, organ failure, pancreatic necrosis, and mortality in acute pancreatitis. HPB Surg, 2013: 367581. 24204087 10.1155/2013/367581 PMC3800571

[33] Rau BM Kemppainen EA Gumbs AA Büchler MW Wegscheider K Bassi C Puolakkainen PA (2007), Beger HG Early assessment of pancreatic infections and overall prognosis in severe acute pancreatitis by procalcitonin (PCT): a prospective international multicenter study. Ann Surg, 245: 745–754. 17457167 10.1097/01.sla.0000252443.22360.46 PMC1877072

[34] Cardoso FS Ricardo LB Oliveira AM (2013). C-reactive protein prognostic accuracy in acute pancreatitis: timing of measurement and cutoff points. Eur J Gastroenterol Hepatol, 25: 784–789. 23492986 10.1097/MEG.0b013e32835fd3f0

[35] Azab B Jaglall N Atallah JP (2011). Neutrophil-lymphocyte ratio as a predictor of adverse outcomes of acute pancreatitis. Pancreatology, 11: 445–452. 21968329 10.1159/000331494

